# Drug-Drug Interaction Predictions via Knowledge Graph and Text Embedding: Instrument Validation Study

**DOI:** 10.2196/28277

**Published:** 2021-06-24

**Authors:** Meng Wang, Haofen Wang, Xing Liu, Xinyu Ma, Beilun Wang

**Affiliations:** 1 School of Computer Science and Engineering Southeast University Nanjing China; 2 Key Laboratory of Computer Network and Information Integration Southeast University Nanjing China; 3 College of Design and Innovation Tongji University Shanghai China; 4 Third Xiangya Hospital Central South University Changsha China

**Keywords:** drug-drug interactions, knowledge graph, natural language processing

## Abstract

**Background:**

Minimizing adverse reactions caused by drug-drug interactions (DDIs) has always been a prominent research topic in clinical pharmacology. Detecting all possible interactions through clinical studies before a drug is released to the market is a demanding task. The power of big data is opening up new approaches to discovering various DDIs. However, these data contain a huge amount of noise and provide knowledge bases that are far from being complete or used with reliability. Most existing studies focus on predicting binary DDIs between drug pairs and ignore other interactions.

**Objective:**

Leveraging both drug knowledge graphs and biomedical text is a promising pathway for rich and comprehensive DDI prediction, but it is not without issues. Our proposed model seeks to address the following challenges: data noise and incompleteness, data sparsity, and computational complexity.

**Methods:**

We propose a novel framework, Predicting Rich DDI, to predict DDIs. The framework uses graph embedding to overcome data incompleteness and sparsity issues to make multiple DDI label predictions. First, a large-scale drug knowledge graph is generated from different sources. The knowledge graph is then embedded with comprehensive biomedical text into a common low-dimensional space. Finally, the learned embeddings are used to efficiently compute rich DDI information through a link prediction process.

**Results:**

To validate the effectiveness of the proposed framework, extensive experiments were conducted on real-world data sets. The results demonstrate that our model outperforms several state-of-the-art baseline methods in terms of capability and accuracy.

**Conclusions:**

We propose a novel framework, Predicting Rich DDI, to predict DDIs. Using rich DDI information, it can competently predict multiple labels for a pair of drugs across numerous domains, ranging from pharmacological mechanisms to side effects. To the best of our knowledge, this framework is the first to provide a joint translation-based embedding model that learns DDIs by integrating drug knowledge graphs and biomedical text simultaneously in a common low-dimensional space. The model also predicts DDIs using multiple labels rather than single or binary labels. Extensive experiments were conducted on real-world data sets to demonstrate the effectiveness and efficiency of the model. The results show our proposed framework outperforms several state-of-the-art baselines.

## Introduction

An increasing amount of research in clinical studies is focusing on drug-drug interactions (DDIs) because the majority of adverse drug reactions (ADRs) occur between pairs of drugs. ADRs may lead to patient morbidity and mortality, accounting for 3% to 5% of all in-hospital medication errors [1]. Furthermore, patients with 2 or more diseases (eg, older adult patients with chronic diseases) have a higher risk of an ADR if they take 5 or more different drugs simultaneously [2,3]. Detecting DDIs based on experimentation is a time-consuming and laborious process for clinicians. This signals the need for a more comprehensive and automated method of predicting unknown DDIs before a new drug can be released.

Traditional experimental approaches in vitro [4], in vivo [5], and in populo [6] focus on small sets of specific drug pairs and have laboratory limitations. Many machine learning approaches, such as similarity or feature-based approaches [7-9], have been proposed to predict DDIs. Recently, several graph neural networks and long short-term memory methods based on knowledge graphs (KGs), such as KG neural network [10] and KG-DDI [11], have significantly outperformed traditional shallow machine learning methods. The superior performance of these proposed methods can be attributed to their use of the prior knowledge and learning of higher-level representations for DDI detection. However, as these approaches only predict binary DDIs or those that have been predefined in structured databases, they may be hampered by robustness caused by data sparsity and vast computation requirements. Although several approaches [12-14] have used natural language processing techniques to extract DDIs from biomedical text, to the best of our knowledge, they have not employed drug KGs to improve performance.

With the increasing emergence of biomedical data, many world-leading biomedical researchers are now focusing on automatically populating and completing biomedical KGs using the huge volume of structured databases and text available to the public. HKG [15], Knowlife [16], and DrugBank [17] are just a few examples. Efforts such as Bio2RDF [18] and Linked Open Drug Data [19] have mapped similar entities in different KGs and built large heterogeneous graphs that contain an abundance of basic biomedical facts about drugs. SPARQL [20], a query language for KGs, supports the retrieval and manipulation of drug-related facts distributed over different KGs. Unfortunately, these biomedical KGs are affected by incomplete and inaccurate data that impede their application in the field of safe medicine development.

Existing KGs already include thousands of relation types, millions of entities, and billions of facts [19]. As noted, KG applications based on conventional graph-based algorithms are restricted by data sparsity and computational inefficiency. To address these problems, graph embedding techniques [9,21-26] based on representation learning for KGs have been proposed that embed both entities and relations into a continuous low-dimensional vector space. Among these methods, translation-based models [9,22,24] are the most simple and effective. Currently, they represent the state-of-the-art in knowledge acquisition and inference and link prediction [9]. In light of these analogies, DDIs can be treated as a category of relations in a drug KG, and KG embedding techniques can be used to predict unknown DDIs. However, most translation-based methods only concentrate on predefined relations or unstructured text and fail to exploit the link between existing relations and rich unstructured text.

Leveraging both drug KGs and biomedical text is a promising pathway for rich and comprehensive DDI prediction, but it is not without issues. Our proposed model seeks to address the following challenges: data noise and incompleteness—real-world KGs are known to be inaccurate, incomplete, and unreliable for direct use; data sparsity—the potential DDI information in both KGs and biomedical text is sparse, and estimating the potential DDIs in such a long-tailed distribution is difficult; computational complexity—undoubtedly, this will be precluded from practice if graph-based algorithms are employed to process large-scale KGs or represent data objects with simple one-hot feature vectors.

Given these challenges, we propose a novel framework called Predicting Rich DDI (PRD). The framework is based on graph embedding techniques and treats specific DDI predictions as a linked prediction process. The proposed framework proceeds as follows: A large, high-quality drug KG is generated from distributed drug resources, which includes data on drug-target interactions, the impact of drugs on gene expression, the outcomes of drugs in clinical trials, and so on. A novel translation-based embedding model embeds the entities and relations in the drug KG into a low-dimensional space, and an autoencoder incorporates the descriptions of the DDIs from biomedical text as representations into the same semantic space. The decoder predicts the corresponding labels for potential DDIs based on the learned embeddings.

To the best of our knowledge, our PRD approach is the first method that is able to predict comprehensive and specific DDIs based on large-scale drug KGs and comprehensive biomedical text on pharmacology and ADRs. Our method further includes a joint translation-based embedding model that encodes the KG and rich DDI information from biomedical text into a shared low-dimensional space. The DDI predictions are then translated into a linked prediction process from the learned embeddings. Extensive experiments on real-world data sets were conducted to evaluate the framework. The results show that the framework can be powerful in predicting rich DDIs and outperforms several state-of-the-art baselines in terms of both capability and accuracy.

## Methods

**Figure 1:**
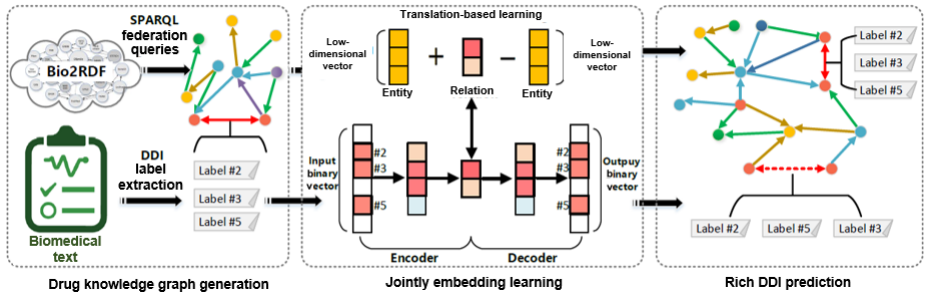
Overview of the framework. DDI: drug-drug interaction.

Figure 1 shows the architecture of the proposed framework. It consists of 3 key phases: drug KG generation, joint embedding learning, and DDI relations prediction.

### Drug KG Generation

A typical KG usually arranges knowledge as a triple set of facts that indicates the relation between 2 entities, and thus comprises a head entity, a relation, and a tail entity. These are denoted as (h, r, t).

First, a basic drug KG is constructed by collecting drug-related entities and relations among these entities. We follow the data model of drug-related extraction settings defined in the work of Kamdar and Musen [27], in which the types of entities or relations are summarized in the fashion depicted in Table 1. Specifically, we use SPARQL federation queries [20] to extract triples that contain 4 types of drug-related entities (*E*_1_~*E*_4_) and 5 types of biological relations (R_1_~R_4_) from a variety of biomedical sources (eg, Bio2RDF [18]). These extracted triples are defined as basic triples in our drug KG according to definition 1: (basic triple) *B* = (*E*, *R*) is a set of basic triples in the form (*h*, *r*, *t*), where *E* = *E*_1_∪ E_2_ …∪ E_4_ is a set of entities; and *R* = *R*_1_ ∪ *R*_2_…∪ R_5_ is a set of relations*, h, t ∈ E,* and r ∈ R*.*

For instance, we can extract “(etanercept, hasTarget, lymphotoxin-alpha)” as a basic triple in our drug KG, which indicates that there is a relationship “hasTarget” linking etanercept to lymphotoxin-alpha, meaning that lymphotoxin-alpha is one of the targets of etanercept.

**Table 1:** Entities and relations of basic triples in Kamdar and Musen [27].

Variable		Entity or relation interpretation
***E***		
	*E_1_*	Drugs
	*E_2_*	Drugs
	*E_3_*	Pathways
	*E_4_*	Phenotypes
***R***		
	*R_1_*	Drug, hastarget, protein
	*R_2_*	Drug, hasenzyme, protein
	*R_3_*	Drug, hastransporter, protein
	*R_4_*	Protein, ispresentin, pathway
	*R_5_*	Pathway, isimplicatedin, phenotype

**Figure 2:**
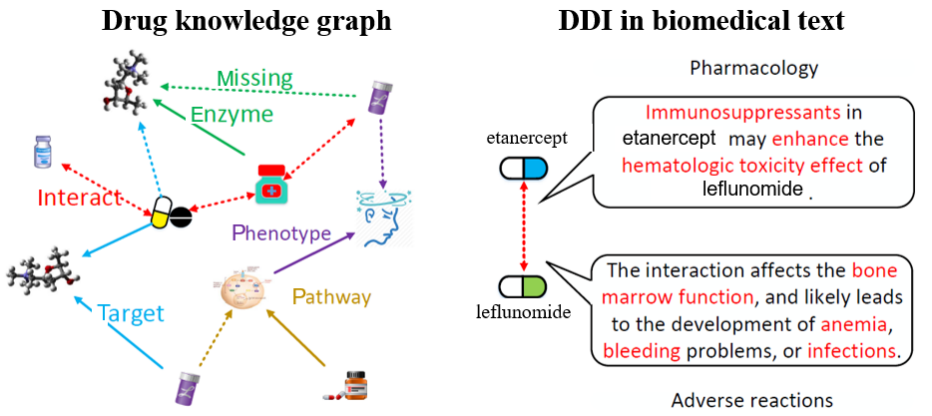
A drug knowledge graph is shown on the left with missing relations represented as dotted lines. There is usually no direct DDI relation between drugs. DDI descriptions from the biomedical text are shown on the right. The words in red represent concerns regarding DDI information in terms of both adverse DDIs and in-depth ways drugs can interact in pharmacology. DDI: drug-drug interaction.

A specific DDI between 2 drugs can be captured by multiple key phrases extracted from biomedical text, as shown in Figure 2. Hence, we collect biomedical DDI text documenting drug pairs (eg, DDI corpus [28], MEDLINE abstracts, and DrugBank DDI documents). We remove all stop words from raw text and use an entity linking method [29] to align the drug names in the biomedical text with the KG. The top-n labels (n=5) are then selected from the biomedical text for each DDI based on the term frequency-inverse document frequency (TF-IDF) features (some other textual features can be used to rank the labels instead).

Based on this, the DDI relations between drug entities are defined as a set of labels rather than as a single label according to definition 2: (rich DDI triple) *T* = (E_1_, *L*) is a set of rich DDI triples in the form (*u*, *l, v*), where *E_1_* is a set of drug entities*;* L is a fixed label vocabulary from biomedical text*;* and u, v ∈ *E*_1_ and *l* = {n_1_, n_2_, …} ⊆ *L* is the set of labels to describe the DDI information.

For instance, the following is an example of a rich DDI triple: (etanercept, {immunosuppressants, enhancetoxicity, anemia, infections}, leflunomide), where “enhancetoxicity” means etanercept can enhance the toxicity of leflunomide. Note that the DDI relations between 2 drugs are bidirectional; hence, our method replaces each rich DDI relation with 2 directed triples of opposing directions in the drug KG.

Formally, the generated drug KG is defined according to definition 3 (drug KG): the drug KG, G, is denoted as (*E*, *B*, *T*), where E = *E*_1_ ∪ *E*_2_…∪ *E*_4_ is a set of entities*, B* is a set of basic triples, and T is a set of rich DDI triples.

### Joint Embedding Learning

KG embedding mainly consists of 3 steps: representation of entities and relations, definition of a scoring function, and encoding of the entity and relation into dense vectors. This section introduces the translation-based KG embedding model that learns representations from the drug KG, G = (*E*, *B*, *T*) and the optimization described in the following sections.

#### Basic Triple Encoder

For a set of basic triples, B, the method aims to encode entities and relations into a continuous vector space. This paper, without loss of generality, uses the bold letters **h**, **r**, **t** to denote the embedding vectors h, r, t. We adopt the translation-based mechanism **h** **+** **r** **≈** **t** to capture the correlations between entities and relations. Translation in this context refers to a translation operation **r** between 2 entity vectors **h** and **t** in the low-dimensional space. We follow the TransR model in Lin et al [22] to represent entities and relations in distinct vector spaces bridged by relation-specific matrices so as to learn more thorough graph representations. Specifically, for each triple, (h*, r, t*) ∈ *B*, h and t are embedded into h, t ∈ R^k^, and r is embedded into *r* ∈ R*^d^*. For each relation *r*, a projection matrix M_r_ ∈ R ^(k×d)^ ×projects entities from the entity space to the relation space. The energy function *z*_bte_ (**h**, **r**, **t**) is then defined as follows:

*z*_bte_ (**h, r, t**) = b_1_ – ‖**hM**_r_ + **r** – **tM**_r_ ‖_(L1/L2)_ (**1**)

where *b*_1_ is a bias constant.

The conditional probability of a triple h, r, t is defined as follows:



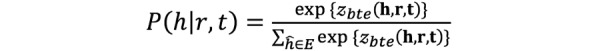
(**2**)


*P*(*t*|*h*, *r*), *P*(*r*|*h*, *t*) can be defined in an analogous manner. The likelihood of observing a triple (h, r, t) is defined as follows:

L(h, r, t) = logP(h│r, t) + logP(t│h, r) + logP(r│h, t) (**3**)

By maximizing the conditional likelihoods of all existing triples in *B*, the objective function is defined as follows:



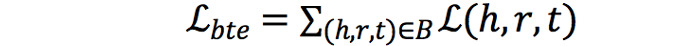
(**4**)


It is worth mentioning that other graph embedding models, such as HOLE [23], can also be easily adopted for basic triple encoding. In the interest of brevity, this paper only explores the effectiveness of TransR.

#### Rich DDI Triple Encoder

The interaction *l* between 2 drug entities, u and v, in rich DDI triples (*u*, *l*, *v*), *∈* *T*, can also be represented as translations in low-dimensional space. We set **u**, **v** *∈* R^k^, **l** *∈* R^d^. The energy function *z*_dte_ (*u*, *l*, *v*) is defined as follows:

*z*_dte_ (**u, l, v**) = b_2_ – ‖**uM**_r_ + **l** – **vM**_l_ ‖_(L1/L2)_ (**5**)

where *b*_2_ is a bias constant and **M**_l_ = R^×^*^d^* is the projection matrix. Following the analogous method in the basic triple encoder, the conditional likelihoods of all existing triples are maximized as follows:



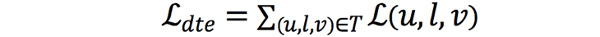
(**6**)


Note, in equation 5, l is the relation representation obtained from *l* = {*n*_1_, *n*_2_,…}. This will be introduced in-depth next.

A deep autoencoder is employed to construct the relation representation *l* *∈* *R*^d^ for a rich DDI triple (*u*, *l*, *v*) ∈ T. Specifically, a DDI relation, *l*, is described by a set of labels *l* = {*n*_1_, *n*_2_,… } *⊆ L*. The corresponding binary vector for *l* is initialized as **s** = 
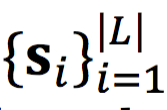
, where **s***_i_* = 1 if *n_i_ ∈ l*, and **s***_i_* = 0 otherwise. The deep autoencoder then takes the binary vector **s** as input and uses the following nonlinear transformation layers to transform the label set into the low-dimensional space R*^k^*:

h^(1)^ = *f*(**W**^(1)^ s + **b**^(1)^)

*h*^(^*^i^*^)^ = *f*(**W**^(^*^i^*^)^*h*^(^*^i^*^–1)^ + b^(^*^i^*^)^), *I* = 2, …, *K* (**7**)

where *f* is the activation function and *K* is the number of layers. Here, h^(^*^i^*^)^, **W**^(^*^i^*^)^, and **b**^(^*^i^*^)^ represent the hidden vector, transformation matrix, and the bias vector in the *i*-th layer, respectively.

There are 2 parts to the autoencoder: an encoder and a decoder. The encoder employs the *tanh* activation function to obtain the DDI relation representation **l** = *h*^(^*^K^*^/2)^. The decoder deciphers the embedding vector of **l** to obtain a reconstructed vector 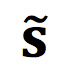. Intuitively, PRD should then minimize the distance 
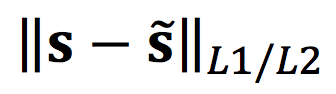
 because the reconstructed vector 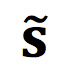 should be similar to **s**. However, the number of zero elements in **s** is usually much larger than that of nonzero elements due to data sparsity. This leads the decoder to tend to reconstruct zero elements rather than nonzero elements, which conflicts with our purpose. To overcome this obstacle, different weights are set for different elements, and the following objective function is maximized:



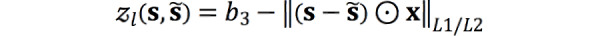
(**8**)


where b_3_ is a bias constant, **x** is a weight vector, and ⊙ is denoted as the Hadamard product. For **x** = 
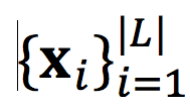
, **x***_i_* = 1, if **s***_i_* = 0, and **x***_i_* = β > 1 otherwise. According to equation 8, the probability of *P* 
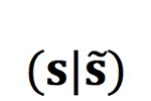
 can be defined as follows:



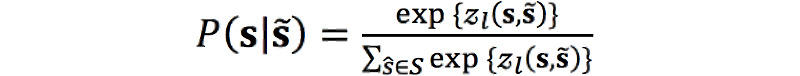
(**9**)


where *S* is the set of binary vectors of all DDI relations. The likelihood of reconstructing the binary vector s of a relation *l* can be defined as follows:


*L*(*l*) = *logP*
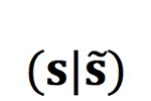
 (**10**)


By maximizing the likelihoods of the encoding and the decoding for all described relations l, the objective function can be defined as follows:



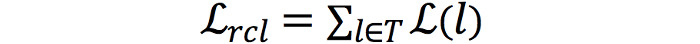
(**11**)


#### Joint Learning and Optimization

The goal of the framework PRD is to not only represent the basic triples (drug KG *B*) but also the rich DDI triples (biomedical text *T*) in a unified joint embedding model. Considering the above 3 objective functions (equations 4, 6, and 11) together, the optimization function is defined as follows:

*O*(*X*) = L*_bte_* + *L_dte_* + L*_rcl_* + *γC*(*X*) (**12**)

where *X* represents the embeddings of entities and relations, and *γ* is a hyper-parameter that weights the regularization factor *C*(*X*), which is defined as follows:


*C*(*X*) = 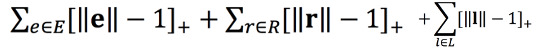 (**13**)


where [*x*]_+_ = *max*(0, *x*) denotes the positive part of *x*. The regularization factor will normalize the embeddings during learning. We adopted the approach by Srivastava et al [30] to prevent deep neural networks from overfitting and used the Adam algorithm [31] to maximize the objective function.

It is impractical to directly compute the normalizers in *P*(*h*│*r*, *t*), *P*(*t*│*h*, *r*), *P*(*r*│*h*, *t*), and *P* 
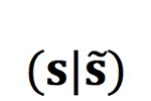
, as calculating them would require summing the complete set of entities and relations. To address this problem, we use the negative sampling method from Mikolov et al [32] to transform the objective functions. Taking *P*(*h*│*r*, *t*) as an example, the following objective function is maximized instead of using its original form:

*logσ*(*z_bte_* (**h**, **r**, **t**))

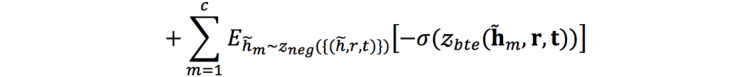
(**14**)


where *c* is the number of negative examples, *σ*(*x*) = 1/(1 + *exp*(–*x*)) is the sigmoid function, 
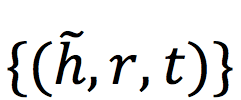
 is the invalid triple set, and *z_neg_* is a function randomly sampling instances from 
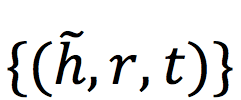
. When a positive triple (*h*, *r*, *t*) *∈ B* is selected to maximize equation 14, *c-*negative triples are constructed by sampling entities from a uniform distribution over *E* and replacing the head of (*h*, *r*, *t*). The objective functions of *P*(*r*│*h*, *t*), *P*(*t*│*h*, *r*), *logP* 
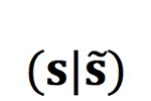
, and *L*(*u*. *l*. *v*) are transformed and maximized in an equivalent manner. Finally, PRD iteratively selects random mini-batches from the training set to learn the embeddings efficiently until convergence.

### DDI Relations Prediction

The DDI prediction task can be defined as a link prediction problem on KG; that is, with the learned deep autoencoder and the embedding vectors of all entities and relations, the framework PRD can leverage the translation mechanism to predict the missing DDI relations between 2 drug entities. To be more specific, given 2 drug entities *u*, *v* *∈* *E*_1_, the following equation predicts the potential relation embedding **l** between **u** and **v.**

**l** = **vM***_l_* – **uM***_l_* (**15**)

Finally, with the decoder part of the learned deep autoencoder, PRD can obtain the label set *l* by decoding the embedding vector l.

## Results

To examines the effectiveness of the DDI prediction framework PRD, we performed 2 types of experiments. First, we compared the performance of our model to several baseline methods on binary-type DDI predictions. We then investigated PRD’s strengths in modeling rich DDI relations between drug entities. The results demonstrate that PRD significantly outperformed the baselines in terms of both accuracy and capability.

### Data Construction

Experiments in this paper were performed on 2 real drug-related data sets, Bio2RDF [18] and DDI Corpus [28].

Bio2RDF (version 4) is an open-source project that provides 11 billion triples from 35 biological and pharmacological KGs across a wide variety of drug-related entities, such as proteins, targets, and diseases. It is accessible online via the SPARQL endpoint.

DDI Corpus (2013 version) is a semantically annotated corpus of documents describing DDIs from the DrugBankdatabase and MEDLINE abstracts. It contains 233 MEDLINE abstracts and 784 DrugBank texts on the DDIs subjects. There are a total of 5021 annotated DDIs in 18,491 pharmacological sentences.

Following the federation queries in Kamdar and Musen [27], we extracted basic triples for our drug KG from 4 different KGs in Bio2RDF: (1) DrugBank [17] provides comprehensive data about drug, disease, and target information; (2) Kyoto Encyclopedia of Genes and Genomes [33] offers pathways, proteins, and drugs information; (3) PharmGKB [34] contains protein-drug-disease relations; (4) Comparative Toxicogenomics Database ([35] provides data about protein interactions and pathway-disease relations.

For the rich DDI triples, we collected 4694 DrugBank DDI sentences about 8197 drugs from the DDI corpus. The top 5 labels from each sentence were selected based on TF-IDF to construct rich DDI triples and build the DDI label vocabulary. To overcome the issue of inconsistent drug names between basic triples and rich DDI triples, we applied the entity linking method [29] to align the drug aliases.

The drug KG we constructed contains 71,460 basic triples, 4694 rich DDI triples, 8197 drug entities, 305,642 other entities, and 1053 distinct labels in the DDI vocabulary.

### Baselines

For the baseline approaches, DDI prediction and state-of-the-art KG embedding methods were used. Three DDI methods were used:

1. Tiresias [8] is a large-scale similarity-based framework that predicts DDIs through link prediction. It takes various sources of drug-related data and knowledge as inputs and generates binary DDI predictions as outputs.
2. Syntax Convolutional Neural Network (SCNN) [36] represents a DDI extraction method based on a SCNN to extract 4 predefined DDI types (ADVICE, EFFECT, INT, and MECHANISM) from the biomedical literature.
3. Multitask dyadic DDI prediction (MDDP) [37] defines the DDI type prediction problem as a multitask dyadic regression problem. It can predict the specific DDI type between 2 drugs.

Two state-of-the-art KG embedding methods were used:

1. TransE [9] is the most representative translational distance model to embed components of a KG, including entities and relations, into continuous vector spaces. These embeddings can also be used for link prediction.
2. TransR [22] shares a similar approach with TransE, but represents entities and relations in distinct vector spaces bridged by relation-specific matrices.

### Evaluation Method and Metrics

Given a drug KG with some DDI relations removed, rich DDI prediction aims to predict the occurrence of DDI relations among drug entities. DDI relations with a rate of 0.3 chosen randomly as the ground truths for the test set were removed, and the remaining KG was used as the training set. We also randomly sampled an equal number of drug pairs with no DDI relations to serve as the negative sample in the test set.

To make an unbiased comparison, we first treated DDI prediction as a binary classification task. Tiresias is already a binary classification model. For SCNN and MDDP, we defined the 2 DDI types as yes and no in the training model. For TransE, TransR, and our PRD method, we concatenated the representations of the entities of a candidate drug pair to form the feature vector and used logistic regression to train classifiers. We then treated multiple DDI type predictions as a multilabel classification task. For Tiresias, SCNN, and MDDP, we used their feature representation methods and adopted one-versus-rest logistic regression to train a multilabel classifier. For TransE and TransR, we separated each training triple (*u*, *l*, *v*) where *l* = {*n*_1_, *n*_2_,…} into several triples (ie, [*u*, *n*_i_, *v*] for *n*_i_ *∈* *l*), which could be directly used to train the models.

We used 10-fold cross-validation on the training set to tune PRD’s embedding model. We determined the optimal parameters using a grid search strategy. The search ranges for the various parameters were as follows: the learning rate λ for the Adam algorithm {0.1, 0.01, 0.001}; γ for the soft constraints {0.1, 0.01, 0.001}; the vector dimension *k* {20, 50, 80, 100}; and all bias constants *b*_1_, *b*_2_, *b*_3_, *c* were 10 to 10. The training instances were conducted over 1000 iterations. The running time per iteration was 391 seconds. The best configurations for the joint model were *λ*=0.001, *γ*=0.01, *k*=100, *b*_1_=5, *b*_2_=5_,_ *b*_3_=1, *c*=10, and *K*=3*,* with *L*_1_ being used as a dissimilarity metric.

We used receiver operator characteristic curves and precision-recall curves to evaluate the proposed method on binary DDI-type predictions. For multiple DDI- type predictions, we followed the setting in TransE [9] and report the 2 measures as evaluation metrics: the average rank of all correct relations (MeanRank) and the proportion of correct relations ranked in top k (Hits@k). The above metrics may be biased for methods that rank other correct labels higher in the same label set. Hence, all other correct labels were filtered out before ranking. The filtered version is denoted as “Filter,” and the unfiltered version is denoted as “Raw.”

### Experiment Results

**Figure 3:**
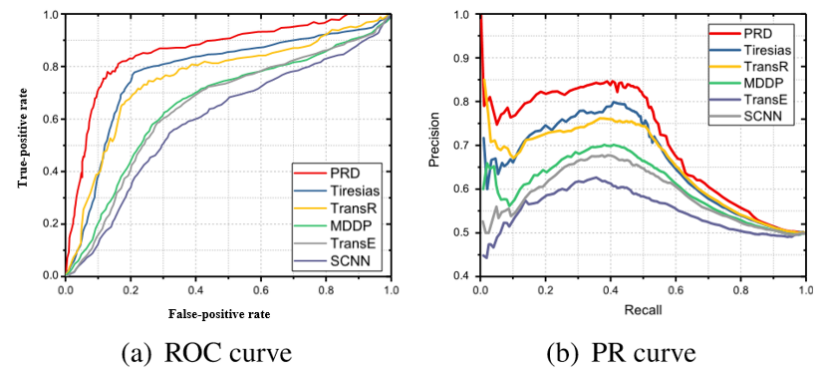
ROC and PR results of binary drug-drug interaction-type predictions. MDDP: multitask dyadic drug-drug interaction (DDI) prediction; ROC: receiver operator characteristic; PR: precision-recall.

As shown in Figure 3a and Figure 3b, the proposed framework PRD outperformed all baselines. In terms of the receiver operator characteristic curve, PRD outperformed Tiresias by 6.69%, TransR by 7.13%, and MDDP and TransE by 12%; meanwhile, SCNN had a relatively low predictive ability. According to the precision-recall curve, PRD learned 14.2% better than did Tiresias (which was at the top among the 3 DDI prediction baselines), 16.8% better than TransR, 21.57% better than MDDP, 25.33% better than TransE, and 37.89% better than SCNN.

Table 2 shows the evaluation results for rich DDI relation predictions according to the different evaluation metrics for both the raw and filter tests.

**Table 2:** Evaluation results for multiple drug-drug interaction relation predictions (×100 for Hits@k).

Framework	Raw					Filter			
	Hits@1^a^	Hits@5	Hits@10	MeanRank^b^	Hits@1		Hits@5	Hits@10	MeanRank
Tiresias	14.23	33.18	50.61	21.89	19.21		45.29	52.94	17.93
SCNN^c^	12.19	26.31	39.02	37.91	16.82		27.03	40.78	37.06
MDDP^d^	20.95	58.66	79.48	13.53	43.19		68.57	84.12	7.85
TransE	26.61	70.23	83.97	8.01	57.88		79.99	87.27	7.02
TransR	31.33	75.80	87.63	6.89	69.58		84.01	89.01	6.25
PRD^e^	45.11	85.57	91.01	6.11	75.11		88.60	92.85	5.45

^a^Hits@x: accuracy of real values contained in the top x rank.

^b^MeanRank: the average rank of all correct relations.

^c^SCNN: Syntax Convolutional Neural Network.

^d^MDDP: multitask dyadic drug-drug interaction prediction.

^e^PRD: Predicting Rich Drug-Drug Interaction.

### Case Study

To further demonstrate PRD’s ability for rich DDI predictions, we selected the drug acetylsalicylic acid (aspirin) as a test case. The DDI predictions and rich labels relations are shown in Table 3. According to the usefulness and diversity of the predicted labels, a professional pharmacist evaluated and annotated the practical useful predictions (labels in italics in Table 3). Observe that both TransR and PRD were able to recommend reasonable DDI labels for the drug interactions, representative of detailed DDI information. However, TransR sometimes recommended similar labels for a specific drug because it is based on a similarity method. Conversely, PRD was able to recommend discriminative labels because it uses a decoder.

**Figure 4:**
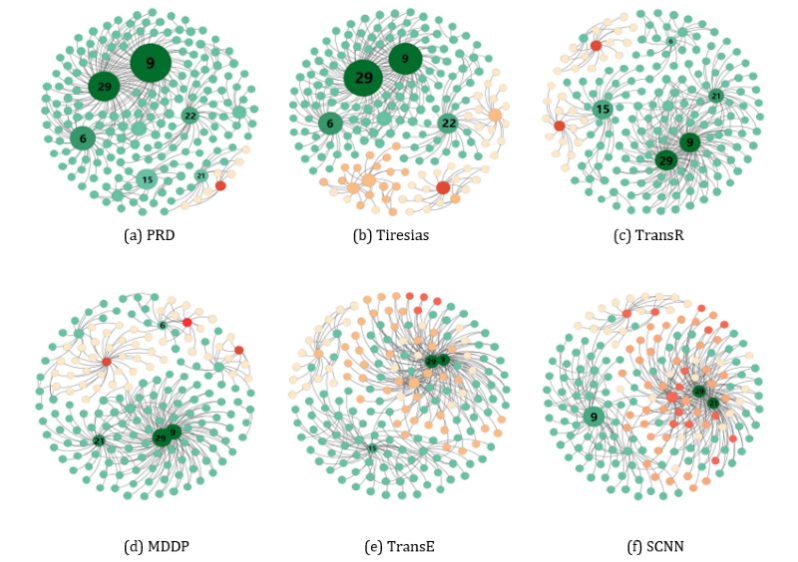
Case visualization of the binary drug-drug interaction-type prediction on a drug-drug interaction network sample. MDDP: multitask dyadic drug-drug interaction prediction; PRD: Predicting Rich Drug-Drug Interaction; SCNN: Syntax Convolutional Neural Network.

We also present a case study to visualize the effectiveness of binary DDI types of prediction on a DDI network sample. We constructed drug-drug networks to indicate whether any 2 drugs would result in a binary DDI. A node in the network denotes a drug. An edge between 2 nodes denotes the existence of a DDI. Intuitively, the more drugs interact, the more risk there is. In the network, the size of the node specifies the degree of risk of a drug. We classified the degree of risk into various levels using different colors (ie, high risk is shown in dark green, and low risk is shown in light green). The red nodes denote forecasting errors of DDI drugs. As shown in Figure 4a to Figure 4f, PRD predicts DDIs mostly accurately. The ID of the drug with a high risk is shown on the node.

**Table 3:** Rich drug-drug interaction predictions for acetylsalicylic acid.

Interacted drug	TransR^a^	PRD^b^
Ibritumomab	*enhance*^c^*adverse*, *toxic*, risk, bleeding	*enhance*, *toxic*, *bleeding*, *platelet*, *antiplatelet*
Alteplase	*enhance*, increase, *adverse*, toxic, effect	*enhance*, *toxic*, *bleeding*, *thrombolytic*, *adverse*
Anistreplase	*enhance*, effect, *thrombolytic*, *agents*, *anticoagulant*	*enhance*, *anticoagulant*, *antiplatelet*, *thrombolytic*, *agents*
Ramipril	*diminish*, *antihypertensive*, effect, treatment, affect	*diminish*, *antihypertensive*, *inhibitor*, *doses*, affect

^a^TransR: a knowledge graph embedding model, which performs translation in the corresponding relation space.

^b^PRD: Predicting Rich Drug-Drug Interaction.

^c^Labels in italics indicate those annotated by a professional pharmacist.

## Discussion

### Principal Findings

PRD achieved a significant improvement over all baselines. Specifically, PRD outperformed MDDP by around 10%. MDDP is currently considered to be the best DDI prediction baseline for multiple DDI type predictions. Tiresias and SCNN performed poorly because they neglect various types of semantic information concerning DDIs. These results demonstrate the effectiveness of PRD to predict rich DDI relations among drug entities.

Compared to TransR and TransE, PRD also performed better, as it incorporates binary DDI types into the relation representation learning and also models multiple DDI labels of a DDI relation simultaneously. This accounts for its promising results in rich DDI prediction.

### Conclusions

PRD is unlike other existing models. Using rich DDI information, it can competently predict multiple labels for a pair of drugs across numerous domains, ranging from pharmacological mechanisms to side effects. To the best of our knowledge, this framework is the first to provide a joint translation-based embedding model that learns DDIs by integrating drug KGs and biomedical text simultaneously in a common low-dimensional space. The model also predicts DDIs using multilabels, rather than single or binary labels. Extensive experiments were conducted on real-world data sets to demonstrate the effectiveness and efficiency of the model. The results show PRD outperforms several state-of-the-art baselines. In future work, we intend to incorporate a convolutional neural network to encode the rich DDI text to improve the performance of the embedding model. Another direction for our research is to have the embedding model consider subgraph features composed in the generated drug KG during learning. This may make it possible to predict DDIs among 3 or more drugs.

## Abbreviations

ADR: adverse drug reaction
DDI: drug-drug interaction
KG: knowledge graph
MDDP: multitask dyadic drug-drug interaction prediction
PRD: Predicting Rich Drug-drug Interaction
SCNN: Syntax Convolutional Neural Network
TF-IDF: term frequency-inverse document frequency
